# Site‐directed mutagenesis in bread and durum wheat via pollination by *cas9*/guide RNA‐transgenic maize used as haploidy inducer

**DOI:** 10.1111/pbi.13415

**Published:** 2020-06-11

**Authors:** Nagaveni Budhagatapalli, Thomas Halbach, Stefan Hiekel, Heike Büchner, Andreas E. Müller, Jochen Kumlehn

**Affiliations:** ^1^ Plant Reproductive Biology Department of Physiology and Cell Biology Leibniz Institute of Plant Genetics and Crop Plant Research (IPK) Gatersleben Germany; ^2^ Strube Research GmbH & Co. KG Söllingen Germany

**Keywords:** *BRI1*, Cas endonuclease, CRISPR, doubled haploid, *SD1*, targeted mutagenesis

Site‐directed mutagenesis facilitates the experimental validation of gene function and can speed up plant breeding by producing new genetic variability or by reproducing previously known gene variants in other than their original genetic backgrounds. However, its application is challenging in wheat owing to high genomic redundancy and highly genotype‐dependent DNA transfer methods (Koeppel *et al*., 2019). In wheat, large chromosomal regions are hardly amenable to meiotic recombination, which limits the potential for trait improvements. The era of transgenesis facilitated the generation of desired traits through the transfer of recombinant DNA into elite backgrounds. This technology, however, is limited by long and costly regulatory evaluation processes owing to publicly overrated method‐specific risks. As another option, the use of meiotically recombinant and genetically fixed doubled haploids proved very useful for accelerating crop improvement (Kalinowska *et al*., 2019). Viable methods of *in planta* haploid induction via uniparental genome elimination are available in species such as *Arabidopsis* through modification of *CENTROMERIC HISTONE 3* (*CENH3*) (Ravi and Chan, 2010), in maize and rice via knockout of a sperm‐specific phospholipase gene (Kelliher *et al*., 2017; Yao *et al*., 2018), and in wheat through intergeneric crossing with maize (Laurie and Bennett, 1988). Haploid induction coupled with site‐directed mutagenesis has previously been reported in *Arabidopsis*, maize and wheat (Kelliher *et al*., 2019). However, in wheat, no unambiguous evidence has been provided yet, considering that a mutated target sequence was shown for just a single event. Furthermore, proof of heritability of site‐directed mutations is still lacking. The present study involves intergeneric pollination of wheat with *cas9*/guide RNA (gRNA)‐transgenic maize to facilitate site‐directed mutagenesis in any wheat germplasm of choice. For exemplification of this principle, new allelic variants were generated for the wheat genes *BRASSINOSTEROID‐INSENSITIVE 1* (*BRI1*) and *SEMI‐DWARF 1* (*SD1*) which are involved in the regulation of plant height.

The present approach relies on the expression of *cas9* and wheat gene‐specific gRNA in maize sperm cells. Therefore, transgenic maize carrying a ubiquitously expressed *GFP* was analysed, and conspicuous fluorescence was found in sperm cells (Figure 1a). Two Cas9/gRNA target motifs for *TaBRI1* and one for *TaSD1* were selected. These proved to be conserved across all two (AABB) or three (AABBDD) homeologues of the target genes in durum and bread wheat, respectively. Corresponding gRNAs were cloned into generic vectors used to transform maize (Budhagatapalli *et al*., 2016). Two hundred maize T_0_ plants carrying wheat target‐specific *cas9*/gRNA‐encoding T‐DNAs were prescreened by qRT‐PCR analysis. Per target motif, five maize transgenics with high *cas9* and gRNA expression were selected for pollination of wheat (Figure 1b). Upon these intergeneric crosses, embryos were rescued *in vitro*. Regenerated wheat plants were then subjected to PCR‐based mutation analysis by Sanger sequencing. For *BRI1* target motif 1, three, two and one mutants were obtained out of 83, 44 and 10 plants in genotypes BW, W5 and D6, respectively. Two plants out of 4 and 3 carried mutations for *BRI1* target motif 2 in genotypes W5 and D7, respectively. In addition, seven mutants for the *SD1* target motif 1 were obtained from 17, 5 and 8 plants in genotypes BW, K15 and S96, respectively (Figure 1c). Subcloning and Sanger sequencing of target motif‐derived amplicons of M_1_ plants indicated that all bread wheat mutants for *BRI1* and *SD1* were invariably homozygous, whereas those in durum genotypes D6 and D7 were chimeric (Figure 1c). This may be due to differences in *cas9* and gRNA expression, the time point of male genome activation and the activity of DNA repair.

**Figure 1:**
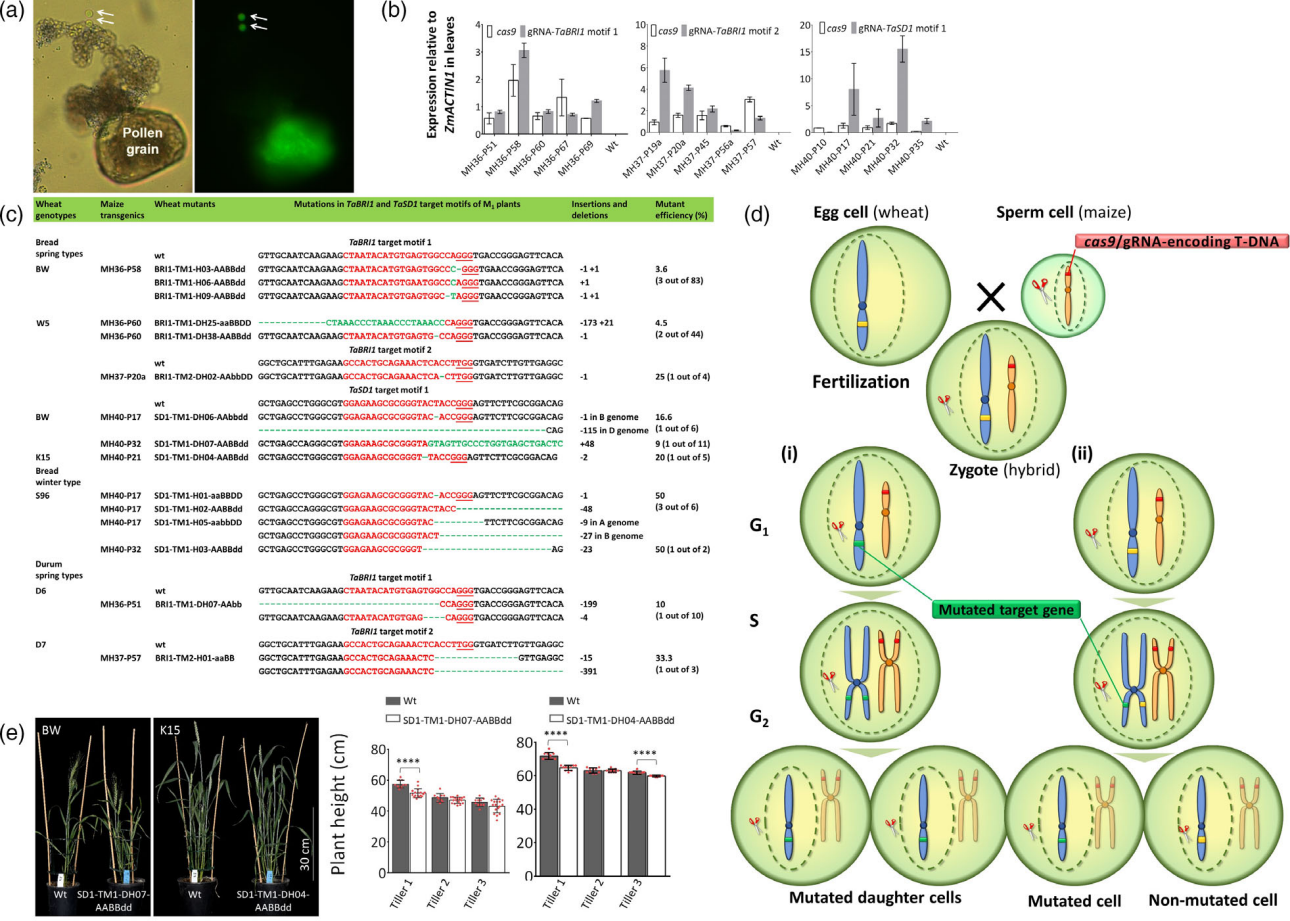
Site‐directed mutagenesis in bread and durum wheat via pollination by *cas9*/gRNA‐transgenic maize. (a) GFP accumulation in maize sperm cells (arrows); bright field (left) and GFP (right) filter images with 200x magnification. (b) Expression of *cas9* and gRNAs. Graphs represent the mean values of *cas9* and gRNA, and error bars represent the standard deviation derived from three replications. (c) The *TaBRI1* and *TaSD1* target motifs are highlighted in red with the protospacer‐adjacent motifs (PAM) being underlined. Mutations are indicated by green font, and the numbers given to the right represent the concerned nucleobases. Mutant efficiency is the proportion of plants with mutations out of the total number of plants analysed. Abbreviations: (H/DH) haploid/doubled haploid, (TM) target motif, (wt) wild type, (+) insertions and (−) deletions. (d) Mutations induced in hybrid zygotes at various phases before and after mitosis. (e) Tiller height of representative doubled‐haploid *SD1* M_2_ plants at anthesis stage. The graph represents the mean height values of the first three tillers from the BW‐wt (*n* = 10), SD1‐TM1‐DH07‐AABBdd mutant (*n* = 17), K15‐wt (*n* = 10) and SD1‐TM1‐DH04‐AABBdd mutant (*n* = 10). Significant differences between mutants and wt counterparts are indicated by asterisks, with **** representing a *P*‐value < 0.0001 according to unpaired *t*‐test; *n* is the number of plants analysed, and error bars represent the standard deviation.

In the present approach, mutations can be induced at various phases before and after the zygote undergoes mitosis (Figure 1d). Mutations induced during G_1_ and early S phase are more likely to occur owing to Cas9 and gRNA molecules pre‐produced in the sperm rather than to zygotic *de novo* transgene expression. Resultant embryos are expectedly non‐chimeric with regard to the induced mutations (Figure 1d‐i). Alternatively, after chromatid duplication, Cas9 may trigger mutations in one chromatid or independently in either of the sister chromatids (Figure 1d‐ii). In this scenario, the daughter cell that has received a mutated wheat chromatid during the first embryonic mitosis itself undergoes S phase, by which the mutated allele becomes genetically fixed across the two sister chromatids, while the other daughter cell has received a non‐mutated or differently mutated chromatid and thus gives rise to a genetically distinct sector. Consequently, embryos formed via mutagenesis during G_2_ phase are expectedly chimeric (Figure 1d‐ii). In the course of initial embryonic cell divisions upon wheat x maize crosses, maize chromosomes are eliminated due to asynchronous processing in terms of DNA replication, condensation and centromere formation (Laurie and Bennett, 1988).

In total, 15 independent target gene‐specific mutants were identified out of 174 wheat plants from which good‐quality Sanger sequences of target motifs had been retrieved. Mutants were obtained in six wheat backgrounds, including the three spring‐type bread wheats BW, W5 and K15, the winter‐type bread wheat S96, and the two durum wheats D6 and D7 (Figure 1c). Mutations were found in all three target motifs addressed (Figure 1c). None of the 15 mutants carried any transgene. Across the genotypes, the efficiency in mutant plant formation ranged from 3.6% to 50% (Figure 1c). The *BRI1* and *SD1* genes are known to play an important role in plant height. Therefore, loss‐of‐function mutants may entirely fail to develop. In addition, knockouts of *BRI1* and *SD1* (*GA20ox*) in *Arabidopsis* lead to male sterility, as they regulate key genes of anther and pollen development (Plackett *et al*., 2012; Ye *et al*., 2010). The haploid plants obtained in the present work were subjected to colchicine treatment. As a result, 7 out of 15 mutants were fertile (Figure 1c). In M_2_, progenies of doubled‐haploid mutants SD1‐TM1‐DH04‐AABBdd of genotype K15 and SD1‐TM1‐DH07‐AABBdd of genotype BW proved to have invariably inherited the very same mutations detected in their M_1_ progenitors (2‐bp deletion and 48‐bp insertion in the D subgenome, respectively) (Figure 1c). These primary mutants were thereby confirmed to be non‐chimeric and true breeding. The M_2_ plants displayed a reduced plant height phenotype. At the anthesis stage, the height of tiller 1 exhibited an average reduction of 6 and 5 cm compared with the wild‐type in genotypes K15 and BW, respectively (Figure 1e). The weak phenotype of these mutants is likely due to the still functional *SD1* homeologues of the A and B genomes which may largely compensate the loss of function of the *sd1* alleles of the D genome.

In conclusion, the principle of haploid induction coupled with site‐directed mutagenesis was exemplified in wheat using the two target genes *BRI1* and *SD1* which control the agronomically important trait plant height. Major advances achieved in this work include reduced genotype dependence of site‐directed mutagenesis in wheat, the opportunity of creating a whole variety of mutations using just one *cas9*/gRNA‐transgenic (pollinator) plant as well as the production of T‐DNA‐free and frequently homozygous M_1_ plants. There is still scope for increasing the efficiency of this approach, for example by stronger transgene expression at the relevant time point or by the development of improved protocols for *in planta* production of doubled haploids.

## Conflict of interest

The authors declare no conflicts of interest.

## Author contributions

J.K. conceived the project concept, and N.B., T.H., H.B. and A.E.M. designed and performed the experiments. N.B. and J.K. wrote the manuscript, and T.H., S.H. and A.E.M. reviewed and edited the manuscript. All authors read and approved the manuscript.
